# Engaging Culturally and Linguistically Diverse Communities to Prepare for Lung Cancer Screening Implementation in Australia: A Qualitative Focus Group Study

**DOI:** 10.1111/hex.70557

**Published:** 2026-01-18

**Authors:** Tescha Nicholls, Sarah York, Nathan J. Harrison, Georgia Bartlett, John Paul Troiani, Biljana Stanoevska, Mei Ling Yap, Joel Rhee, Rachael H. Dodd, Henry M. Marshall, Annette McWilliams, Emily Stone, Leissa Pitts, Nicole M. Rankin

**Affiliations:** ^1^ Centre for Health Policy, Melbourne School of Population and Global Health University of Melbourne Melbourne Victoria Australia; ^2^ Ramsey Health Care, Border Cancer Hospital Albury New South Wales Australia; ^3^ Flinders Health and Medical Research Institute, College of Medicine and Public Health Flinders University Adelaide South Australia Australia; ^4^ Illawarra Shoalhaven Local Health District Warrawong New South Wales Australia; ^5^ Collaboration for Cancer Outcomes, Research and Evaluation, Ingham Institute UNSW Sydney Liverpool New South Wales Australia; ^6^ George Institute for Global Health UNSW Sydney Barangaroo New South Wales Australia; ^7^ Liverpool and Macarthur Cancer Therapy Centres Western Sydney University Campbelltown New South Wales Australia; ^8^ Sydney School of Public Health, Faculty of Medicine and Health The University of Sydney Camperdown New South Wales Australia; ^9^ Discipline of General Practice, School of Clinical Medicine, Faculty of Medicine and Health UNSW Sydney Kensington New South Wales Australia; ^10^ The Daffodil Centre, A Joint Venture Between The University of Sydney and Cancer Council NSW, Faculty of Medicine and Health The University of Sydney Camperdown New South Wales Australia; ^11^ University of Queensland Thoracic Research Centre and Department of Thoracic Medicine The Prince Charles Hospital Chermside Queensland Australia; ^12^ Department of Respiratory Medicine Fiona Stanley Hospital Murdoch Western Australia Australia; ^13^ Faculty of Health and Medical Sciences University of Western Australia Perth Western Australia Australia; ^14^ Department of Thoracic Medicine and Lung Transplantation St. Vincent's Hospital Sydney Darlinghurst New South Wales Australia; ^15^ School of Clinical Medicine, Faculty of Medicine and Health UNSW Sydney Kensington New South Wales Australia

**Keywords:** acceptability, culturally and linguistically diverse communities, early detection, focus groups, implementation, lung cancer screening, multicultural

## Abstract

**Introduction:**

Lung cancer is the number one cause of cancer death worldwide, including in Australia, where more than 15,000 cases were diagnosed in 2024. The Australian National Lung Cancer Screening Programme commenced in July 2025. Unique barriers to participation in existing cancer screening programmes exist for culturally and linguistically diverse (CALD) communities. Little Australian research has engaged these communities to gauge their acceptability and readiness for the implementation of lung cancer screening (LCS). The aims were to identify barriers and enablers to LCS implementation and determine views about LCS acceptability and feasibility.

**Methods:**

Community‐based participatory research principles were used to collaboratively engage CALD stakeholders. The research team worked with highly experienced programme managers, multicultural health workers and bilingual facilitators/translators (‘leaders’) who provide support and education to Arabic‐speaking/Lebanese, Macedonian, Vietnamese and Italian communities. Leaders used culturally appropriate methods to recruit participants and deliver focus groups. Verbatim transcripts were analysed using a coding framework based on the Health Equity Implementation Framework.

**Results:**

Seven focus groups were conducted either face to face (*n* = 5) or online (*n* = 2) with 57 participants across New South Wales, Australia. Six groups were delivered in community languages. LCS was generally perceived as acceptable across all focus groups. Key enablers towards engagement were understanding the importance of screening for early detection, provision of interpreters and easily accessible locations. Key barriers were financial and language barriers, poor experiences with other cancer screening programmes, concerns about tobacco‐related eligibility criteria and cancer‐related stigma.

**Conclusions:**

This qualitative study provides crucial foundational evidence from four culturally diverse Australian communities about barriers and enablers to inform Programme implementation. The findings are highly relevant to other jurisdictions that may be preparing for LCS implementation or need to modify programmes to engage culturally diverse populations to screen.

**Patient or Public Contribution:**

Members of the public were directly involved as participants in this research. Multicultural health workers and bilingual facilitators/interpreters who work with CALD communities were involved in all phases of the study design and implementation, including recruitment, development of semi‐structured focus group guide, focus group facilitation and reviewing the manuscript.

## 1 Introduction

Lung cancer is the leading cause of cancer‐related death worldwide, including in Australia [1], where the 5‐year survival rate is 26% [2]. Most people (> 42%) are diagnosed with advanced disease stage [3]. Early detection via lung cancer screening (LCS) in high‐risk populations presents a clinically effective, evidence‐based intervention for people with significant tobacco exposure; in Australia, this represents 8.3% and 28% of the general population, respectively [4].

Randomised controlled trials of low‐dose computed tomography (low‐dose CT) demonstrate a pooled lung cancer‐specific mortality benefit of 21% [5]. Worldwide, about 14 national and jurisdictional LCS programmes have been implemented with significant clinical outcomes already reported [3]. The focus of research has shifted to understanding implementation complexities and challenges [6, 7]. Numerous barriers to LCS engagement have been identified, including lack of awareness (of lung cancer and of screening), access challenges, cost concerns, fear and stigma, lack of shared decision‐making and challenges in identifying high‐risk individuals [8, 9].

Strategies for overcoming implementation barriers include investment in pre‐implementation consultation and engagement with priority populations [10]. In Australia, this includes culturally and linguistically diverse (CALD) communities [11] who are under‐represented in research. Evidence shows that people from CALD backgrounds diagnosed with lung cancer experience higher mortality rates, attributed to a lower likelihood of appropriate and timely care and greater likelihood of being diagnosed with later‐stage disease [12, 13, 14]. Differences in diagnosis, treatment and survival outcomes also exist across some CALD groups [15, 16]. Differences are attributed to factors including cultural, behavioural and social influences, family histories, use of health services and underlying health conditions. It is well established that disparities in health outcomes across population groups reflect power imbalances in social and political constructs that enable systemic discrimination [17, 18].

The Australian population is highly diverse and multicultural. People from some CALD communities report difficulties accessing the Australian healthcare system due to unfamiliar structures, communication difficulties, and views of health that do not align with Western care approaches [13, 19]. These difficulties are mirrored by variations in cultural competency among healthcare providers and by systemic racism and discrimination that continue to affect the Australian healthcare system [13, 18, 19, 20]. Unequal access to healthcare services necessitates equity‐focused research, addressing institutional and societal level change, rather than solely focusing on individual behaviour change. Hence, directly engaging with CALD communities and exploring preferences for LCS is urgently needed [15, 16].

The Australian Government requires evidence that proposed screening programmes are ‘clinically, socially, legally and ethically acceptable to health professionals, consumers and the Australian public’ and should ‘promote equity of and access to screening of the entire target population’ [11] prior to endorsement. The Australian Medical Services Advisory Committee (MSAC) [21] recommended the creation of the Australian National Lung Cancer Screening Programme (hereafter ‘the Programme’) in May 2023. The Programme commenced on 1 July 2025.

Evidence about LCS acceptability and feasibility in the Australian setting was gathered during a National Enquiry [22], explored with International Lung Cancer Screening Trial (ILST) participants [23, 24] and by our team, who have published formative research from the perspectives of healthcare providers, policy makers [25, 26, 27] and tobacco control and smoking cessation experts [28]. However, little is known about the views of people from CALD communities regarding LCS.

The aim of this study was to explore how CALD communities perceive LCS, to identify barriers and enablers to the implementation of LCS in the Australian setting and to gauge how acceptable and feasible [29] the Programme would be within these communities.

## 2 Methods

### 2.1 Study Design and Context

This qualitative study used community‐based participatory research principles [30] to conduct in‐language focus groups with Arabic‐speaking, Lebanese, Macedonian, Vietnamese and Italian communities between December 2022 and December 2023. It was conducted in New South Wales (NSW), Australia's most populous state of ~8.1 million people, where 30% speak a language other than English at home [31]. An estimated 17% of all cancer diagnoses in NSW occur in this priority population [32]. At the time of study inception (May 2019), there was no organised LCS programme in Australia. Focus group topics and discussions were thus framed in the context of a hypothetical or ‘potential’ LCS programme [25, 26]. Participants' perceptions and views were sought in anticipation of potential policy change. However, the recommendation for the Programme was announced [21] prior to some focus groups being conducted (see Table 1).

**Table 1:** Overview of focus groups.

Location	Participant group	Language	Date	Meeting type	Participants
WSLHD^a^	Arabic	Arabic	22 March 2023	F2F	10
ISLHD^b^	Macedonian	Macedonian	27 March 2023	F2F	9
SWSLHD^c^	Arabic	Arabic	11 July 2023	F2F	10
SWSLHD^c^	Lebanese	English	2 August 2023	Zoom	6
SWSLHD^c^	Vietnamese	Vietnamese	14 September 2023	Zoom	8
ISLHD^b^	Italian	Italian	23 November 2023	F2F	5
ISLHD^b^	Arabic	Arabic	16 December 2023	F2F	11

*Note:* Shading indicates focus groups that occurred after the MSAC recommendation for the Programme was announced [23].

^a^ Western Sydney Local Health District.

^b^ Illawarra Shoalhaven Local Health District.

^c^ Southwestern Sydney Local Health District.

### 2.2 Recruitment

The sample comprised adult participants from CALD communities in two metropolitan areas of NSW with high population proportions of people born overseas [33]. Inclusion criteria were that participants identified as culturally diverse and were migrants from non‐Anglophone countries, or were born in Australia and were first‐, second‐ or third‐generation children of migrants. Current or former smoking status was not a criterion for participation. The authors approached experienced programme managers, multicultural health workers and bilingual facilitators/interpreters (‘leaders’) who work with CALD communities and established relationships with leaders in three local health districts. Focus group participants were identified by leaders through existing connections with health education and community groups. Those who were interested in the topic were invited to participate. All participants received an AUD$50 gift voucher as an acknowledgement of their time.

### 2.3 Focus Group Procedures and Processes

Focus group procedures adhered to best practice guidance [34]. Prior to each focus group, a project coordinator conducted briefing sessions with leaders to provide background information on lung cancer and LCS. Guidance was given on collecting informed consent and distributing participant surveys as well as reviewing and refining a draft semi‐structured discussion guide.

Each focus group was facilitated by a bilingual leader with experience in health‐related qualitative data collection. A research team member attended focus groups to provide clarification regarding LCS if requested by the leader and to take notes about topics. In some groups, a bilingual translator was present in addition to the facilitator and provided translations to the research team members to facilitate group communication. All focus groups were conducted in‐language except one, which was conducted in English at the request of participants.

Each group began with an informational video on LCS developed in the UK for the National Health Service to provide an overview for participants [available from https://www.youtube.com/watch?v=U3oirXkufno&t=1s]. A brief introduction from an Australian general practitioner (GP) was recorded to reinforce that LCS was not locally available. The video was professionally translated into community languages by an accredited translation company.

A semi‐structured guide (Data S1) was used in all focus groups based on the team's previous research [25, 26, 27]. It covered topics including investigating awareness of lung cancer and LCS, barriers and enablers, feasibility and acceptability, perceptions about smoking cessation support, co‐design, messaging and awareness campaigns. Focus group procedures and question guide were tested and modified following a pilot focus group with three people living with a lung cancer diagnosis (data not included).

Participants were asked to complete a brief self‐report survey prior to focus groups, including demographic information, the Single Item Literacy Screener to measure health literacy [35] and items about lung cancer and LCS knowledge and attitudes [36].

### 2.4 Data Analysis and Interpretation

All focus groups were audio‐recorded. In‐language recordings were transcribed into English by either a professional translation service or the focus group facilitator (who had professional translation accreditation). The audio recording for the focus group conducted in English was checked by a team member to ensure transcription accuracy. As a result of translation processes, participant quotes cannot be aligned to individual demographic characteristics (e.g., age or gender).

Transcripts were analysed using a thematic analysis approach [37] and codes were mapped to the Health Equity Implementation Framework (HEIF) [38]. The HEIF features six domains which influence implementation: (i) the characteristics of the intervention or *innovation*; (ii) the *clinical encounter* between provider and patient; (iii) *patient factors* and (iv) *provider factors* that affect engagement with intervention; (v) factors at the organisational‐level or *inner context* and (vi) social, cultural and environmental factors of the *outer context*. These domains are highly relevant as they encourage an equity‐based lens to be applied to all facets of LCS that may impact CALD communities' capability and willingness to engage.

An analytic codebook was developed through collaborative coding and discussion among four authors (T. N., N. J. H., G. B. and N. M. R.). Four transcripts were coded independently by two authors (T. N., N. J. H. or G. B.) with discussion and comparisons to identify potentially relevant codes and incorporate additional insights. One author (T. N.) coded all remaining transcripts using the finalised codebook. Microsoft Excel was used to manage the coding process. Codes were mapped to the HEIF domains. Those with similar patterns of meaning were then grouped to generate candidate themes. Themes and sub‐themes were developed by T. N., N. J. H. and G. B., and refined via discussion with N. M. R.

Ethics approval was obtained through the South‐Western Sydney Local Health District Ethics Committee (2021/ETH00826).

## 3 Results

### 3.1 Sample Characteristics

Fifty‐seven participants from Arabic‐speaking, Lebanese, Macedonian, Vietnamese and Italian communities took part in seven focus groups (range *n* = 5–11 participants/group). All groups were facilitated and conducted in community languages, except one conducted in English. Five of the focus groups were conducted face‐to‐face in community venues, and two online.

Participants' demographic information is shown in Table 2. Most participants were aged over 60 (54.4%) and 63.2% were female. Of the 53 participants who reported on their country of birth, 90.6% reported having migrated from other countries, and 9.4% were born in Australia.

**Table 2:** Self‐reported demographic information for focus group participants.

Characteristic	*n* (%)
Age (*N* = 57)	
30–49	11 (19.3)
50–59	15 (26.3)
60–74	26 (45.6)
75–84	5 (8.8)
Gender (*N* = 57)	
Male	21 (36.8)
Female	36 (63.2)
Country of birth (*N* = 53)	
Iraq	10 (18.9)
Lebanon	9 (17.0)
Macedonia	9 (17.0)
Vietnam	8 (15.1)
Australia	5 (9.4)
Italy	4 (7.5)
Sudan	4 (7.5)
Libya	2 (3.8)
Iran	1 (1.9)
Palestine	1 (1.9)
Languages spoken at home^a^ (*N* = 53)	
Arabic	27 (50.9)
Macedonian	9 (17.0)
Vietnamese	8 (15.1)
English	6 (11.3)
Italian	4 (7.5)
Fur	2 (3.8)
Kurdish	1 (1.9)
Assyrian	1 (1.9)
Lebanese	1 (1.9)

*Note:* The number of participants that answered each question is indicated where relevant, reflecting missing data.

^a^ More than one language could be chosen and totals sum to > 100%.

Table 3 presents data about participants' awareness of lung cancer and LCS, past smoking history, participation in cancer screening programmes and general health literacy. Over a third of participants (37.5%) always or often needed assistance reading instructions in English. Most had never smoked (67%) or had previously smoked (18%). About half (48%) had taken part in organised cancer screening programmes and about one‐fifth of participants were aware of LCS prior to the focus groups.

**Table 3:** Responses to survey questions answered prior to focus groups participation.

**Survey item**	***N* (%)**
Have you, or any friends, or family members who are close to you ever been diagnosed with lung cancer? (*N* = 54)	
No	48 (88.9)
Yes, someone close	6 (11.1)
Yes, self	0 (0)
Yes, self and someone close	0 (0)
Yes, prefer not to say who	0 (0)
Have you ever taken part in a cancer screening programme? e.g., BreastScreen, bowel cancer or cervical screening? (*N* = 54)	
Yes	26 (48.1)
No	28 (51.9)
Not sure	0 (0)
Do you smoke any tobacco products? (*N* = 55)	
No, I have never smoked	37 (67.2)
Not now, but I used to smoke	10 (18.1)
Yes, I smoke occasionally	5 (9.1)
Yes, I currently smoke	2 (3.6)
Never, exposure to passive smoke	1 (1.8)
How often do you need to have someone help you when you read instructions, pamphlets or other written material from your doctor or pharmacy in English? (*N* = 56)	
Never	10 (17.9)
Rarely	8 (14.3)
Sometimes	17 (30.4)
Often	10 (17.9)
Always	11 (19.6)
How often do you need to have someone help you when you read instructions, pamphlets or other written materials from your doctor or pharmacy in your own language? (*N* = 56)	
Never	24 (42.9)
Rarely	14 (25.0)
Sometimes	10 (17.9)
Often	5 (8.9)
Always	3 (5.4)
Have you heard anything about screening for lung cancer? (*N* = 56)	
No	35 (62.5)
Yes	11 (19.6)
Don't know	10 (17.9)
Do you think that lung cancer screening is a good idea? (*N* = 54)	
Yes	52 (96.3)
No	0 (0)
Don't know	2 (3.7)
If you were invited to check whether you could have lung cancer screening, would you take up the offer? (*N* = 56)	
Yes, definitely	29 (51.8)
Yes, probably	22 (39.3)
No, probably not	3 (5.4)
Not sure	2 (3.6)
No, definitely not	0 (0)
If lung cancer is detected early, what do you think a person's chance of surviving is? (*N* = 56)	
Excellent	7 (12.5)
Very good	11 (19.6)
Good	20 (35.8)
Fair	9 (16.1)
Poor	1 (1.8)
Very poor	0 (0)
Not sure	8 (14.3)
How often do you worry about your chance of getting lung cancer? (*N* = 56)	
Never	7 (12.5)
Occasionally	11 (19.6)
Sometimes	17 (30.4)
Often	7 (12.5)
Very often	6 (10.7)
I don't know	8 (14.3)

*Note:* Minor differences in total percentages due to rounding.

### 3.2 Findings

The results section is structured under headings representing HEIF domains (top‐level headings), followed by key themes within domains. Illustrative quotes are described using community group designation and edited for conciseness. Table 4 presents a summary of themes mapped against the HEIF. As LCS was not available in Australia when participants were discussing a potential programme, *provider factors* were not considered relevant and instead coded to ‘clinical encounters’.

**Table 4:** Summary of Health Equity Framework Domains and themes developed during analysis and interpretation.

Health Equity Implementation Framework domain	Definitions of domains	Themes
Innovation	Characteristics of the LCS programme	Positive perspectives about LCS
		Negative perspectives about LCS
		Suggestions for LCS implementation
Clinical Encounter	Interaction between provider and patient directly related to LCS discussions	Role of the GP
		Receptiveness to smoking cessation support
Patient Factors	Characteristics of the patient affecting engagement with LCS	Motivators towards participation
		Disinclination towards participation
Inner Context	Organisational‐level factors affecting engagement with LCS	Access to interpreters
		Financial barriers
		Waiting times for appointments and results
Outer Context	Social, cultural and environmental factors affecting engagement with LCS including experiences and perceptions of other cancer screening programmes	Perceptions of organised cancer screening programmes
		Cancer screening not offered in countries of origin
		Awareness of LCS, lung cancer, and risk factors
		Stigma

*Note:* The Provider Factors domain refers to characteristics of the provider affecting engagement with an intervention; however, given that these discussions referred to a potential LCS programme, provider factors were less salient, and data were not mapped to this domain.

#### 3.2.1 Domain 1: The Innovation

##### 3.2.1.1 Positive Perspectives About LCS

Positive views about LCS were endorsed in all focus groups. Initial reactions included that participants thought it was ‘necessary’ (Italian participant) and ‘a great initiative’ (Lebanese participant). This enthusiasm extended to requests to expedite the implementation timeline in three focus groups.

Positive views often reflected participants' perceptions of early detection for increased lung cancer survival and improved health outcomes more broadly. This was discussed in relation to previous experiences with friends or family who received late diagnoses, including for cancers, and for whom curative treatment was unavailable.

I believe that this is very beneficial because early detection significantly increases the chances of successful treatment. (Vietnamese participant)

A minority of participants shared that they were supportive of LCS implementation overall, even if, personally, they would refrain from screening:

I don't want to participate in that, but I want a [health] centre for that to be opened to help. (Macedonian participant)

Participants in one group emphasised that co‐design approaches should be integrated into LCS programme design, stating: ‘A lot of time we have to co‐design later, it's an afterthought. It's so important to have it factored in’ (Lebanese participant).

##### 3.2.1.2 Negative Perspectives About LCS

Whilst negative perspectives were less prominent, some participants raised concerns about LCS eligibility criteria targeting age and smoking history. Reflecting personal anecdotes of friends or family, this included concerns about restrictions on people without cigarette smoking history who could still develop lung cancer.

Anyone should have an opportunity to get tested for lung cancer, regardless of if they are smokers. (Macedonian participant)

Some participants noted that waterpipe tobacco smoking (shisha/hookah) and other tobacco product use were common in some Arabic‐speaking and Lebanese communities. They questioned whether this would be included in the LCS eligibility criteria. Others raised concerns about potential side effects of LCS, including a perceived risk of radiation exposure and queried whether isolation was required after a low‐dose CT scan. Adverse experiences often informed similar concerns of subsequent investigation:

I had a friend who did an endoscopy, and she spent 1 month unable to eat or drink. (Arabic‐speaking participant)

##### 3.2.1.3 Suggestions for LCS Implementation

When asked about future implementation, participants from all focus groups stressed the importance of ensuring LCS information and educational campaigns were available in a variety of community languages. It was emphasised that translated resources would facilitate LCS participation in CALD communities. As two participants reflected: ‘it's better in your language, more important than English’ (Italian participant) and ‘you will do it if it is in Arabic’ (Arabic‐speaking participant).

Participants were asked about their preference for assessing their eligibility for LCS. An emphasis on language and comprehension was reported across all focus groups. Table 5 summarises the options discussed with quotes that illustrate a range of views. For instance, some participants stated a preference to complete eligibility forms in their primary language at home with their family, while others were concerned they would not understand and would prefer to complete this with their GP. Completion of digital eligibility forms was considered a significant barrier to access, particularly for older participants.

**Table 5:** Opinions on offered means of completing eligibility forms for LCS.

Options to complete eligibility form	Illustrative quote supporting the option	Illustrative quote challenging the option
Individually with a doctor or healthcare worker	He [doctor] will be able to understand me better than if I am writing it by myself. (Arabic‐speaking participant)	…my doctor, he is always in a hurry. (Macedonian participant)
At home with family	To complete the form at home with my family, in Macedonian it's better with the family. (Macedonian participant)	Maybe the person doesn't know what is required or doesn't have enough information [to be able to complete the form alone]. (Arabic‐speaking participant)
Electronic forms	English‐speaking people probably like to do it online. (Lebanese participant)	Impossible. (Italian participant) Not us, not at our age. (Italian participant)

Participants identified a wide range of information requirements to support decision‐making about LCS, including risks and benefits of screening, costs, booking appointments and processes, as well as medication contraindications. This extended to understanding the frequency of repeated low‐dose CT scans and investigation/treatment processes following a screen‐detected finding.

A variety of awareness‐raising strategies were discussed. Table 6 summarises unprompted suggestions. Participants discussed the in‐language video presentation at group commencement and most expressed positive reactions, for example, that it was ‘very good, clear [and] very well explained’ (Macedonian participant). For all strategies, presenting information in community languages was a key recommendation; however, there were mixed views about the most effective strategy:

I think with multicultural people, it's better if they see something on TV because if they get something in the mail, they're likely just to put it aside. (Lebanese participant)

When people see something on TV, they forget. It's better with a letter. (Macedonian participant)

**Table 6:** Participant suggestions for strategies about how to communicate LCS with CALD communities.

Suggested communication strategies	Preferences stated by focus groups	Illustrative quote
Video		It's better to watch a video. (Italian participant)
Targeted written material (e.g., letters)		With a letter would be best. (Macedonian participant)
TV advertising in community languages		Having ads on national TV will reach a wider audience, and everyone can catch the message. (Vietnamese participant)
Group events at community venues (e.g., churches)		I would organise focus groups like this, and it would spread more widely. (Vietnamese participant)
Brochures or flip charts		One of the things that worked a lot in this space for us with the bowel screening, the flip chart that was put [out] by the Cancer Institute. (Lebanese participant)
Doctor‐led communication		Communication with your doctor… the family doctor is supposed to make the person aware. (Arabic‐speaking participant)
Radio announcements (in community languages)		I believe that the media is the most important thing, whether it is television or radio. (Arabic‐speaking participant)
Posters on public transport		Posters using electronic screens, during the drive, the driver sees the advertisement. (Arabic‐speaking participant)
Social media		I think definitely a social media platform. (Lebanese participant)

*Note:* Shaded cells indicate how frequently each strategy was suggested across focus groups (total *n* = 7).

When asked about access to low‐dose CT screening, participants stated widespread support for access via general practice referral. However, there was variation in perceptions about access via mobile screening. The latter option referenced Breast Screen mobile vans that are available in parts of Australia. Positive views about the mobile screening were reducing physical barriers such as lack of transport or for people with mobility restrictions. In the Macedonian focus group, concerns were raised about privacy (e.g., ‘it will be public; anyone can see you’) as well as low‐dose CT quality and safety (e.g., ‘not very safe… not very accurate’). Other strategies suggested to reduce physical barriers included providing taxi vouchers or organising carers to support people with a disability.

#### 3.2.2 Domain 2: The Clinical Encounter

Data mapped to the clinical encounter domain largely applies to the role of GPs in the Australian context.

##### 3.2.2.1 Role of the GP

GPs were perceived as both a potential enabler and barrier towards LCS engagement. Participants expressed a high level of trust in their GPs, and believed they should be responsible for initiating and engaging in discussions about LCS:

Definitely your GP… should be saying ‘Hey, I'm going to refer you to do this. (Lebanese participant)

However, participants in most groups, particularly those without a regular GP, were concerned about GPs being the sole way to access LCS. Some raised that GPs could refuse to provide a referral or act as a gatekeeper if people were unaware of LCS, noting that the topic may not be raised during appointments due to time constraints:

If you don't have information, the doctor is your only avenue. (Macedonian participant)

they [doctors] don't explain many things… today [it] is all ‘quick, quick!' (Italian participant)

##### 3.2.2.2 Receptiveness to Smoking Cessation Support

Most participants were highly receptive to the idea of smoking cessation support being offered during LCS clinical encounters. Some suggested it should only be mentioned ‘in passing’ as there was a perception that discussing cessation could deter those not interested in quitting smoking from screening.

I think it will be off putting… I've got an 83‐year‐old mum and she's a very heavy smoker…. when you tell her to stop smoking, it's like a big deal. It's a big no… I feel like for some people it will be off‐putting. (Lebanese participant)

Discussion noted that smoking was a social norm in some communities and could impact willingness to engage in smoking cessation support:

…culturally because our community, it's part of the social gatherings… it's just very acceptable. (Lebanese participant)

#### 3.2.3 Domain 3: Patient Factors

Cancer screening was discussed more generally when describing the factors that might affect LCS engagement. Patient factors were captured as motivators and a sense of capability to take part in screening or beliefs hindering participation.

##### 3.2.3.1 Motivators Towards Participation

Valuing one's health as well as understanding and believing in the importance of screening were strong determinants of participants' motivation to engage with LCS. Many participants indicated they actively prioritise their health.

I know it [cancer screening] is very important, my health—it comes first. (Arabic‐speaking participant)

Reasons reported for engaging with and valuing cancer screening programmes were reflected in statements from Arabic‐speaking participants: ‘to have peace of mind’, ‘to avoid the disease’ and to ‘make sure that there is nothing wrong’. Participants from three focus groups identified that fear about lung cancer may also act as a motivator for screening:

You would get scared and check yourself. (Macedonian participant)

##### 3.2.3.2 Disinclination Towards Participation

Participants discussed potential reasons not to screen, including fatalistic beliefs, with one participant noting: ‘if I was going to die, I would have died’ (Lebanese participant). These sentiments presented as deeply ingrained beliefs that could foster reluctance to engage with LCS and were linked to disinclination towards preventive health behaviours: ‘if it's not broke, then we don't need to fix it’ (Lebanese participant) or ‘if you fear something, you will get it’ (Arabic‐speaking participant). One participant noted: ‘The culture that we all came with, because we are Iraqi… we do not go to the doctor unless we are very sick’ (Arabic‐speaking participant).

Other perceived reasons for potential LCS non‐participation included: ‘fear that they find something’ (Italian participant), a general ‘stubbornness’, ‘lack of concern’ or otherwise unwillingness to engage with health services, including that ‘men often don't go to the doctor…’ (Vietnamese participant). Participants also identified that younger and healthier individuals may be less willing to overcome barriers to engagement, saying they may ‘…not stick around for a doctor's referral… [because] people easily disregard or ignore the symptoms’ (Vietnamese participant).

#### 3.2.4 Domain 4: Inner Context

Themes in this framework domain included access to interpreters, financial barriers and waiting times for appointments and results. These were generally viewed as fundamental considerations for the feasibility of programme implementation.

##### 3.2.4.1 Access to Interpreters

In three focus groups, participants shared previous experiences where interpreters were unavailable to attend medical appointments or the challenges faced when having to rely on telephone‐based interpreter services.

…an interpreter should have been there for my husband, we should have had an interpreter, but he didn't show up…. (Italian participant)

Participants suggested that overall improvements need to be made to the availability of interpreter services in healthcare settings, including a potential LCS programme. They indicated that interpreters could facilitate LCS engagement by increasing participants' understanding.

##### 3.2.4.2 Financial Barriers

Cost was identified as a prominent barrier to LCS engagement and was discussed at length; the focus groups were held at a time when there was uncertainty about what Medicare (Australia's universal health coverage) would fund beyond the costs of low‐dose CT scans. Thus, participants identified potential costs of GP appointments to obtain a scan referral as one concern:

Some people have to pay from their own pocket to see the doctor, the GP, especially now most doctors don't bulk bill… a visit to the doctor can cost between 70 and 75 dollars. (Arabic‐speaking participant)

Concerns about cost extended to specialist appointments for the purpose of follow‐up tests, with participants stating: ‘a visit to the specialist costs us 440 dollars’ (Arabic‐speaking participant), and that associated treatments could be even more expensive. These discussions included choice of healthcare providers and services under Medicare and whether insurance coverage would be needed for attending specialist appointments in private facilities. Participants identified service availability (regardless of insurance coverage) of primary care and radiology providers would be a potential enabler towards engagement with LCS.

…you need to have a choice. If you can't go private, you should be able to have it public. (Italian participant)

Participants in some groups thought that LCS participation should incur no out‐of‐pocket costs at all: ‘It would be easier to participate if it's free’ (Macedonian participant).

##### 3.2.4.3 Waiting Times for Appointments and Results

Waiting times required to see a GP were noted as an organisational‐level barrier with potential to limit engagement. For example, participants in the Vietnamese group thought that lengthy wait times may be a pertinent barrier for those perceived as not motivated (described as ‘lazy’ [Vietnamese participant]) or who are not experiencing symptoms. Conversely, one participant raised the concern about extended waiting times for follow‐up/repeat screening through mobile cancer services and assumed faster access to fixed‐site services for a potential LCS programme.

#### 3.2.5 Domain 5: Outer Context

##### 3.2.5.1 Perceptions of Organised Cancer Screening Programmes

Participants reported widespread awareness of and participation in current organised cancer screening programmes (i.e., breast, bowel and cervical cancers). Awareness of opportunistic screening for melanoma and prostate cancer was also noted. Participants' awareness and experiences of screening informed their perceptions of LCS. Some participants expressed positive perceptions of early detection through screening programmes.

I know bowel screening really saves lives… cervical screening saves women's lives… it's really exciting… that the government is approved to do a national programme like this [LCS]. (Lebanese participant)

Negative experiences with other screening programmes were assumed to impact potential LCS programme engagement. Difficulties completing at‐home bowel cancer screening were noted, including one participant who found the test to be: ‘very confusing… [and] I had to really read the instructions…’ (Lebanese participant). Other negative experiences included a lack of awareness about screening processes, minimal follow‐up communication, including after tests without significant findings and potential risks involved with invasive tests.

##### 3.2.5.2 Cancer Screening not Offered in Countries of Origin

In most focus groups, participants expressed gratitude that organised cancer screening programmes were available in Australia, unlike in their countries of origin. Many appreciated the opportunity to engage, as one participant said:

We are lucky to be in a country like Australia, where all of this is offered to us… it's not like that in Lebanon. (Lebanese participant)

##### 3.2.5.3 Awareness of LCS, Lung Cancer and Risk Factors

There was low awareness of the concept of targeted screening for asymptomatic individuals. Some participants stated they were unaware prior to the focus groups of ways to test or treat lung cancer, while others described requesting opportunistic screening from primary care in response to heightened concerns. They suggested the strategy of developing ‘lung disease checklists’ to encourage appropriate help‐seeking for those with respiratory symptoms.

The causal link between tobacco smoking and increased lung cancer risk was noted, and groups generally recognised that most lung cancer cases were smoking‐attributable, for example, ‘when we hear about lung cancer, it is from smoking… the most’ (Arabic‐speaking participant). Several participants noted the harms of passive cigarette smoke exposure and erroneously thought that quitting could substantially reduce or even ‘cure’ lung cancer risk. Further misperceptions regarding smoking included that quitting smoking could be *harmful* for those with heavy smoking histories:

Some smokers [if] they smoked for years, and stopped smoking suddenly, they will have residue in their lungs that will make them worse. (Arabic‐speaking participant)

Views were also mixed on the relative risks of waterpipe tobacco smoking, with one Lebanese participant describing community misperceptions: ‘shisha is healthier, and it doesn't affect you, and it doesn't give you cancer’ (Lebanese participant). In most groups, participants discussed additional modifiable and non‐modifiable factors perceived to increase lung cancer risk including diet, alcohol consumption, physical inactivity, stress, genetics, occupational (i.e., chemicals, dust) and environmental hazards (e.g., radiation, air pollution, war).

##### 3.2.5.4 Stigma

Cancer‐related stigma was discussed, with most prominence in the Arabic‐speaking and Lebanese groups. In the former, participants described immediately thinking about death in relation to cancer, and that lung cancer cannot be treated or cured:

The word cancer is fear, it is terror. When you hear the word ‘cancer’, you get scared. (Arabic‐speaking participant)

In the Lebanese group, there was a greater emphasis on cancer being a form of ‘punishment’, reflecting on what were described as community beliefs:

If you have cancer… you've done something in your life… you've been punished. (Lebanese participant)

Rather than stigma of cancer itself, another group talked of the belief that ‘the more tests… the more screenings you do, the worse it is for you’ (Italian participant). In contrast, some participants shared a belief that cancer‐related stigma in their community had decreased over time and that stigma would be less of a barrier to LCS programme engagement:

There is no shame. It used to be in the past, not now. It's better to talk about it, to seek treatment. (Macedonian participant)

## 4 Discussion

The aim of this study was to investigate the feasibility and acceptability of LCS for CALD communities in metropolitan NSW, Australia. We present novel findings, being the first Australian study to report on views of LCS prior to Programme commencement from a number of CALD communities' members. The findings highlight that an agile and responsive Programme must consider in‐language resources, affordability, accessibility and community views about cancer to overcome significant barriers to participation. The themes show the variety of perceptions across the sample of Arabic‐speaking, Lebanese, Macedonian, Vietnamese and Italian communities. The findings highlight a need for widespread engagement of CALD communities to create evidence about preferences for LCS implementation and the multiple strategies that will increase participation and access. Initiatives beyond the translation of resources and information materials are required to overcome the documented inequities of the Australian healthcare system [15], including stigma and fear, which were clearly expressed as barriers to engagement.

The HEIF framework [38] enabled an equity‐focused lens to the study. Although barriers were identified for the innovation of low‐dose CT screening and participant‐level behaviours, many barriers discussed relate to general health‐system barriers that could result in inequitable health outcomes. With regards to the innovation, awareness‐raising methods strongly support in‐language communication strategies and developing resources specifically for priority CALD communities (see Table 6). A recent review found varying results in improving behavioural intentions to undergo screening using a variety of awareness‐raising methods [39]. While our study did not seek to reach consensus on optimal methods, the findings strongly support that multiple strategies are needed to reach eligible members across diverse CALD communities.

Regarding accessibility of low‐dose CT screening, participants expressed diverse views about the acceptability of mobile screening vans. It was acknowledged that mobile vans could improve access [40] and increase visibility of services [26, 41]. Participants in our study raised concerns similar to those documented about privacy, cleanliness and crowding [41]. UK pilot LCS programmes have reported that community‐based mobile vans targeting high‐risk populations are effective at engaging ‘hard‐to‐reach’ populations [42, 43]. In the Australian Programme, mobile screening trucks have been funded for rural and remote community outreach [44], who may face additional access needs not identified in this metropolitan sample. Mobile screening is considered a viable option by health professionals, policymakers and programme managers to improve access and equity [26]; however, it is unlikely to be made available to metropolitan participants [45].

The role of the GP was a key interaction as part of the ‘clinical encounter’. Participants expressed contrasting views of GPs as both an enabler and barrier to screening, as described in previous research, whereby a GP's recommendation for screening may act as both a ‘cue to action’ [46, 47, 48] or as a deterrent [49]. Previous Australian‐based research has shown varying levels of trust in GPs across different CALD populations [50]. While study participants generally indicated high levels of trust [50], they did nominate time constraints, lack of a regular GP and difficulties booking appointments as barriers to LCS uptake [47, 51]. Introducing strategies that reduce time burden, such as community‐based education classes, could increase engagement and increase overall acceptability [52]. In addition, health system and policy changes to increase accessibility of primary care services, which incentivise longer appointments and improve continuity of care for CALD communities, may also facilitate greater Programme uptake.

Overall, the findings indicate that patient factors act as enablers towards LCS engagement and strongly align with previous research with culturally diverse racial and ethnic minority communities in the US. These studies showed cancer screening as a form of self‐care [46] and respect for one's health should prompt screening [49], as well as decreasing concerns and improving treatment outcomes [51].

Fatalistic beliefs about cancer and death were frequently discussed and featured in the published literature, often linked to religious or cultural beliefs [13, 48, 53, 54, 55]. In the analysis, fatalistic beliefs were coded in a different domain from social attitudes towards cancer stigma. However, we note an interplay across these issues, as stigma and fear of cancer in some CALD communities (included in the ‘outer context’ theme) have been identified as a barrier to LCS participation. Previous research has identified that fatalistic beliefs about cancer diagnosis or survival were generally viewed as unchangeable [13, 48, 53, 54, 55]. Interventions to encourage shifting beliefs are in development, with limited research published specifically on LCS [56]. The role of the social stigma that exists in some communities towards people diagnosed with cancer was discussed. Similar research with specific CALD communities has found cancer may be considered a taboo topic in some cultures and not discussed outside of immediate family, if at all [55, 57]. The concept of self‐blame from having smoked cigarettes was less prevalent in our study compared to previous ones [46, 58]. This may reflect that most participants in our study reported no or limited smoking history. These beliefs further isolate those experiencing symptoms and discourage screening behaviour, reinforcing these fatalistic ideas due to late diagnosis [13].

The organisational‐level factors or ‘inner context’ that may affect LCS engagement include language translation. While language barriers [48, 59, 60, 61] are frequently described as a patient‐level deficit [18], our findings identify a shift to an equity‐informed perspective advocating for change at the organisational level of healthcare. The provision of high‐quality interpreter services or bilingual health workers, who are conversant and knowledgeable about cultural and religious beliefs, may increase the acceptability and feasibility of LCS in some CALD communities but require investment from healthcare institutions and the government. While reliance on GPs, nurses and family members who are capable of conversing in multiple languages could help overcome some language barriers, these are not sufficient to overcome the entrenching of further inequities in LCS programmes [13, 49, 57, 61].

Financial barriers were prominent across the focus groups and have similarly featured across the LCS literature [46, 47, 62]. Previous research has identified that individuals in some CALD communities may delay preventive healthcare due to cost [49, 63, 64]. Out‐of‐pocket costs limit access to GPs for many Australians, including members of CALD communities, potentially perpetuating inequities and acting as a barrier to LCS.

Many participants in our study had relatively high awareness of existing cancer screening programmes. In contrast to many previous studies that maintained a narrower focus on the screening programme of interest [46, 47, 49, 51, 59, 60, 65], data generated in our study could aid greater understanding of the intersections across cancer screening programmes. Strategies that leverage discussion at other screening appointments could positively influence engagement and enhance LCS uptake.

Some study participants expressed taking active steps to address or mitigate various lung cancer risk factors (e.g., refraining from smoking). However, it was unclear during focus groups if participants considered these lifestyle adjustments sufficient to reduce their risk of lung cancer, and thus would refrain from screening, or if LCS would be welcomed as an additional method of prevention. Low health literacy and English language proficiency have previously been associated with lower rates of cancer screening uptake [62, 66]. Misunderstandings or lack of awareness about when LCS should occur (i.e., prior to symptom presentation), causes of and risk for lung cancer in people who do not smoke, were a concern. This highlights a need for education and LCS awareness‐raising campaigns. As discussed above, no educational method has been identified as superior, and multiple methods may be required to disseminate information [25]. However, it is important to recognise that education alone may not translate to higher uptake of screening [67, 68].

### 4.1 Strengths

A key strength of the study was a robust approach of conducting focus groups in community languages, led by expert facilitators who could elicit and probe for community‐relevant perceptions that would not have been possible if groups had been conducted in English. A high level of engagement was achieved by leaders engaging participants who were first‐generation migrants, who spoke languages other than English in their homes, and for many, required some level of assistance with reading instructions, pamphlets or other written materials in English, from healthcare providers. The authors attribute this success to leveraging community‐based participatory research principles. We collaboratively engaged leaders in the methodological development, recruitment, data collection and analysis, some of whom joined the authorship team. These leaders were from three local health districts with high numbers of CALD populations. Employing an equity‐focused implementation framework for analysis was another strength, as most studies do not and is informative for other jurisdictions considering LCS implementation and seeking to increase CALD communities' participation.

### 4.2 Limitations

Selection bias may be present, as some participants were already engaged with community groups and may have had more interest in health‐focused issues. Our sample does not reflect the full diversity of Australian CALD communities, including variations in language, ethnicity, religion, migration experiences, and experiences with healthcare discrimination in the Australian healthcare system. These factors may affect LCS engagement in ways not likely to be fully captured by this sample. We also acknowledge there are unique determinants to screening uptake for CALD communities in other Australian jurisdictions. Nonetheless, this study provides foundational knowledge that can guide future research and co‐design efforts to engage with CALD communities.

A significant proportion of study participants (67.2%) had never smoked and would be ineligible for the Programme [45]. Thus, the sample may not represent all the views of individuals who are eligible for LCS in Australia. We did not check whether participants were eligible for Medicare benefits or how this might influence their views of LCS. While conducting focus groups in‐language is a key strength, our team has limited ability to decipher nuances within the translated texts by cross‐checking with audio recordings. We sought to overcome this by having a team member observe the groups. The team also regularly engaged with leaders to debrief and add richness to the data analysis.

### 4.3 Implications for Policy, Practice and Research

There is a growing body of research about co‐designing of health programmes with community stakeholders to close evidence‐practice gaps about equity [6, 69, 70]. The project builds the foundation for positively engaging in relationships with leaders in community health settings, paving the way for co‐design of implementation strategies, such as community awareness campaigns and shared decision‐making tools. These strategies may maximise participation in an LCS programme among communities who will most benefit [71]. The research outcomes can be translated to inform policy decisions related to Programme promotion, the design and dissemination of resources, specific support for CALD or other priority populations, and future adaptations to programme eligibility.

## 5 Conclusion

This study explored the acceptability and feasibility of LCS for CALD communities in Australia. Key findings highlighted that LCS is largely perceived as acceptable. Multiple barriers that may inhibit engagement from CALD communities, but these are not insurmountable. The design and implementation of strategies at organisational and health system levels is required to improve equitable outcomes. Inclusive engagement with CALD communities is likely to improve the reach and quality of the Australian Programme. The study findings will be highly informative for other jurisdictions preparing for LCS implementation where there is a need to modify programmes to engage culturally diverse populations in LCS.

## Author Contributions

**Tescha Nicholls:** investigation, formal analysis, data curation, writing – original draft, writing – review and editing, methodology, visualisation, resources. **Sarah York:** methodology, data curation, project administration, resources, supervision, investigation, writing – review and editing. **Nathan J. Harrison:** methodology, writing – original draft, investigation, validation, formal analysis, data curation, writing – review and editing. **Georgia Bartlett:** methodology, data curation, writing – original draft, investigation, writing – review and editing, formal analysis, project administration, visualisation. **John Paul Troiani:** methodology, data curation, investigation, writing – review and editing, formal analysis, resources. **Biljana Stanoevska:** methodology, data curation, investigation, formal analysis, resources, writing – review and editing. **Mei Ling Yap:** conceptualisation, methodology, investigation, funding acquisition, writing – review and editing, formal analysis. **Joel Rhee:** conceptualisation, funding acquisition, writing – review and editing, methodology, formal analysis. **Rachael H. Dodd:** methodology, investigation, writing – review and editing, formal analysis. **Henry M. Marshall:** conceptualisation, funding acquisition, writing – reivew and editing, methodology, formal analysis. **Annette McWilliams:** conceptualisation, funding acquisition, writing – review and editing. **Emily Stone:** conceptualisation, writing ‐ review and editing, funding acquisition. **Leissa Pitts:** methodology, resources, supervision. **Nicole M. Rankin:** conceptualisation, methodology, data curation, investigation, validation, formal analysis, supervision, resources, project administration, visualisation, funding acquisition, writing – review and editing, writing – original draft.

## Ethics Statement

Ethics approval was obtained through the South‐Western Sydney Local Health District Ethics Committee (2021/ETH00826). All study participant information sheets were translated into community languages and provided to the participants prior to the focus groups. Support to understand the documents was available from the study interpreters and/or translated documents where necessary. Participants provided informed written consent using an approved form or verbally at the commencement of focus groups using a script approved by the Ethics Committee.

## Consent

All patients gave written consent to participate in this research project.

## Conflicts of Interest

J.R. has received honorarium from Merck, Sharpe & Dohme and Pfizer for advising, chairing and speaking at clinician education events. The other authors declare no conflicts of interest.

## Patient or Public Contribution

Members of the public were directly involved as participants in this study. Multicultural health workers and bilingual facilitators/interpreters who work with CALD communities were involved in all phases of the study design and implementation, including recruitment, development of semi‐structured focus group guide, focus group facilitation and reviewing the manuscript. We acknowledge the legacy of Ms Carolyn Riordan (deceased) who contributed to development of the research questions for this study (one of four projects conducted within the grant).

## Supporting information

CALD LCS Appendix FG Discussion Guide 13092025.

## Data Availability

Data cannot be shared publicly due to privacy or ethical restrictions. Public availability may compromise the identities of individual participants and/or community group members. Reasonable requests for data may be sent to nicole.rankin@unimelb.edu.au or alternatively to South‐Western Sydney Local Health District Ethics Committee via SWSLHD-Ethics@health.nsw.gov.au (Project 2021/ETH00826).
