# Supplementary material for: Engaging Culturally and Linguistically Diverse Communities to Prepare for Lung Cancer Screening Implementation in Australia: A Qualitative Focus Group Study
Source: Health Expect. 2026 Jan 18;29(1):e70557. doi: 10.1111/hex.70557 (PMC12813417; doi:10.1111/hex.70557)
Supplement: Supplementary file 1 — CALD LCS Appendix FG Discussion Guide 13092025. [file HEX-29-e70557-s001.docx]

# **Appendix 1:**

**Focus Group Discussion Guide**

1. **Today we’d like to hear your views about lung cancer screening.**

**Please could you briefly introduce yourselves, and if you’d like to do so, tell us whether you have any experience with lung cancer?**

*Prompts*

- If someone says the words ‘lung cancer’ what are your first thoughts or reactions?
- Have you heard about lung cancer? What have you heard? What do you understand cancer to be?

*******Presentation of LCS informational video******

1. **Cancer screening involves simple tests that look for early signs of cancer, or the conditions that cause cancer. Screening tests can find cancer before you can see or feel any changes to your body. When you find cancer early, it is easier to treat successfully. What types of cancer screening do you know about?** *Possible answers may include: breast screening, bowel screening and cervical screening.*

Additional prompts

- Have you had any experiences with cancer screening that you would like to share?
- What do you think are the main reasons people might choose not to participate in cancer screening?

1. **Before seeing the video today, had you heard anything about lung cancer screening in Australia?**

Additional prompts

- If yes, what have you heard?

1. **Do you think we should have a lung cancer screening program in Australia?**

Additional prompts

- If yes, why? / If no, why not?
- Is there anything that you think might put people off from participating in lung cancer screening?

1. **How would you like to be invited to participate in lung cancer screening?**

Additional prompts

- Would you like to receive an invitation letter in the post? See a television advertisement? Be invited to screen by your doctor? See advertisements for screening online/via social media?
- Which of these methods (e.g. personal letter, tv campaign, online messaging, discussion with doctor) do you think would interest the most people in screening?

1. **How would you like to access lung cancer screening?**

Additional prompts

- Would you like to be referred to by your GP?
- Would you like to attend a mobile screening van (similar to current Breast Screen vans)?
- Would you be comfortable attending for a lung cancer screening scan at a hospital or private radiology service?

1. **What information would like to have before deciding about whether to have lung cancer screening?** *Possible answers may include: information about what screening is, what will happen at the screening appointment, what will it cost, where can I have the screening test done, when and how will I get the results, what will happen after I get the results?*

Additional prompts

- How would you like information about lung cancer screening to be presented to you? Would you like to read a poster or brochure/booklet? Would you like to watch a video?

1. **To find out if you are eligible for lung cancer screening, you may be asked a short set of questions about your risk factors. Risk factors may include age, history of smoking, family history. How would you prefer to complete these questions?**

Additional prompts:

- Would you like to complete these questions with your GP? At home in your own time? Online? On a mobile app?
- What are the main reasons you may want to complete these questions on a mobile app? *Possible answers might include - it’s quick and easy, don’t have to make appointment with GP, can complete it my own time*
- What are the main reasons you may not want to complete these questions on a mobile app? *Possible answers may include – I have difficulty with phones/technology, would prefer help with questions from GP/nurse*

1. **Is there anything that might make it easier for you or a family member to take part in lung cancer screening?** *Possible responses may include – more information about screening, help with travel to screening site, mobile screening unit that comes to me, the appointment is at a time that best suits me, the screening is of no/low cost to me, a mobile screening van comes to a location close to me.*

1. **Is there anything that might make it harder for you or a family member to take part in lung cancer screening?** *Possible responses may include - concerns about what the scan involves, transport to and from the scan, appointment times, out of pocket costs (including travel costs), being unwell with other illnesses.*

1. **Are there any specific cultural considerations that might influence whether you or a family member may or may not take part in lung cancer screening?**

1. **What do you think about offering a quit smoking program (which may include Nicotine Replacement Therapy) at the same time as lung cancer screening?**

Additional prompts

- Is this a good idea?
- Why/Why not?

1. **What are your overall thoughts on the lung cancer screening video from the UK that you were shown at the beginning of the focus group?**

Additional prompts:

- Was the information clear?
- Was it easy to understand?
- Did you like hearing from a patient?
- Was the information about screening benefits and harms useful?
- Would this video influence your decision to participate in lung cancer screening?

1. **Is there anything else you would like to say on this topic before we finish up?**
